# Comparing Open-Source Toolboxes for Processing and Analysis of Spike and Local Field Potentials Data

**DOI:** 10.3389/fninf.2019.00057

**Published:** 2019-07-30

**Authors:** Valentina A. Unakafova, Alexander Gail

**Affiliations:** ^1^Cognitive Neurosciences Laboratory, German Primate Center, Göttingen, Germany; ^2^Primate Cognition, Göttingen, Germany; ^3^Georg-Elias-Mueller-Institute of Psychology, University of Goettingen, Göttingen, Germany; ^4^Bernstein Center for Computational Neuroscience, Göttingen, Germany

**Keywords:** spike data, LFP, toolbox, MATLAB, open-source, Python, dimensionality reduction, GLM

## Abstract

Analysis of spike and local field potential (LFP) data is an essential part of neuroscientific research. Today there exist many open-source toolboxes for spike and LFP data analysis implementing various functionality. Here we aim to provide a practical guidance for neuroscientists in the choice of an open-source toolbox best satisfying their needs. We overview major open-source toolboxes for spike and LFP data analysis as well as toolboxes with tools for connectivity analysis, dimensionality reduction and generalized linear modeling. We focus on comparing toolboxes functionality, statistical and visualization tools, documentation and support quality. To give a better insight, we compare and illustrate functionality of the toolboxes on open-access dataset or simulated data and make corresponding MATLAB scripts publicly available.

## 1. Introduction

Analysis of spike and local field potential (LFP) data is an essential part of neuroscientific research (Brown et al., 2004; Stevenson and Kording, 2011; Mahmud and Vassanelli, 2016). There are many already implemented open-source tools and toolboxes for spike and LFP data analysis. However, ascertaining whether functionality of the toolbox fits users' requirements is in many cases time-consuming. Often neuroscientists are even not aware that some functionality is already implemented and start writing their own scripts from scratch which takes time and is error-prone. We aim to provide a practical guidance for choosing a proper toolbox on the basis of toolbox functionality, statistical and visualization tools, programming language, availability of graphical user interface, support and documentation quality. Compared to the existing reviews (Ince et al., 2009, 2010; Ince, 2012; Mahmud and Vassanelli, 2016; Timme and Lapish, 2018), we

- include in the comparison important toolboxes and tools not covered by earlier reviews (e.g., Brainstorm, Elephant and FieldTrip);
- compare in detail common and discuss unique functionality of toolboxes;
- compare and illustrate functionality of the toolboxes on open-access datasets (Lowet et al., 2015; Lawlor et al., 2018; Perich et al., 2018) and simulated data. For readers' convenience we make the corresponding MATLAB scripts publicly available^1^
- overview specialized tools for dimensionality reduction and generalized linear modeling as they are widely used in neuroscientific research (Truccolo et al., 2005; Cunningham and Byron, 2014);
- provide information about documentation and support quality for the toolboxes;
- indicate bibliometric information^2^: while popularity among users alone does not guarantee quality, it can be an important indicator that toolbox's functions are easy-to-use and have been tested.

### 1.1. Scope

We include into our comparison major open-source^3^ toolboxes (see Table 1) for spike and LFP data processing and analysis which have a valid link for downloading, documentation, scientific paper describing the toolbox's features or corresponding method, and which were updated in the last 5 years. In Table 1 we provide a summary of the toolboxes we consider, we list all toolboxes with a brief description in alphabetical order in section 7 with paper reference and downloading link. As a brief introduction to the toolboxes listed in Table 1, Brainstorm (Tadel et al., 2011), Chronux (Bokil et al., 2010), Elephant (Yegenoglu et al., 2017), FieldTrip (Oostenveld et al., 2011), and Spike Viewer (Pröpper and Obermayer, 2013) are toolboxes for the advanced analysis and visualization of electrophysiological data including EEG, MEG, spike, and LFP data, whereas SPIKY (Kreuz et al., 2015) toolbox is focused on the spike data analysis and on monitoring spike train synchrony, and gramm (Morel, 2018) is a visualization toolbox for publication-quality plots of complex data including neural recordings. Note that in our comparison we focus on experimental data following a trial-based approach.

**Table 1:** Features of open-source toolboxes regarding graphical user interface (GUI), visualization tools (VIS), Import/Export of spike and LFP data in various file formats, e.g., recorded with different software/hardware, principal programming language, availability of documentation (DOC), number of citations, and support by updates at least once per year.

**Toolbox, version**	**GUI**	**VIS**	**Import/Export**	**Language**	**DOC**	**Cited**	**Support**	**License**
Brainstorm 3.4	+	+	+	MATLAB	+	>**1,000**	+	GNU GPLv3
Chronux 2.12 v03	−	+	+	MATLAB	+	>**300**	In part	GNU GPLv2
Elephant v0.6.0	−	−	+	Python	+	<30	+	BSD
FieldTrip 23.11.18	−	+	+	MATLAB	+	>**3,000**	+	GNU GPLv3
gramm 2.25	−	+	−	MATLAB	+	<30	+	MIT
Spike Viewer 0.4.2	+	+	+	Python	In part	<30	+	BSD
SPIKY 3.0	+	+	−	MATLAB	In part	<30	In part	BSD
				Python				

*Bold values indicate the number of citations higher than 90*.

We have not listed in Table 1 toolboxes FIND (Meier et al., 2008), infotoolbox (Magri et al., 2009) and STAtoolkit (Goldberg et al., 2009), since they are not available under the links provided by the authors (accessed on 27.03.2019); toolboxes BSMART (Cui et al., 2008), DATA-Means (Bonomini et al., 2005), MEA-tools (Egert et al., 2002), MEAbench (Wagenaar et al., 2005), sigTOOL (Lidierth, 2009), SPKTool (Liu et al., 2011), STAR (Pouzat and Chaffiol, 2009), since they have not been updated during the last 5 years (since 2008, 2005, 2007, 2011, 2011, 2011, and 2012, correspondingly); toolbox SigMate (Mahmud et al., 2012) since it is a beta version; and toolbox OpenElectrophy (Garcia and Fourcaud-Trocmé, 2009) which is not recommended for new users by the toolbox authors^4^.

### 1.2. Documentation/Support

We have indicated “In part” in Documentation column for Spike Viewer and SPIKY since, compared to other toolboxes from Table 1, they do not provide a description of input parameters for most of the functions. This complicates understanding of implementation details for programming-oriented users that use only a part of the toolbox functionality in their analysis workflow. gramm toolbox specifies function input parameters not in code comments but in separate documentation file^5^. Considered version of Elephant only provides a getting started tutorial with more tutorials to be added^6^. Chronux and SPIKY (MATLAB version) toolboxes are not uploaded to GitHub or other public version control systems, which prevents tracking version differences and smoothly reporting bugs (Python version of SPIKY is on GitHub^7^).

### 1.3. Import/Export

Elephant and Spike Viewer are using Neo Python package^8^^,^^9^ (Garcia et al., 2014) with support of many spike and LFP data formats. Brainstorm, Chronux and FieldTrip support working with several spikes file formats (e.g., Blackrock, CED, Neuralynx, Plexon etc.)^10^ as well as working with data from MATLAB workspace or stored as .mat files. SPIKY and gramm support working with data from MATLAB workspace; SPIKY also supports working with data stored in .mat and .txt file formats. In Table 2 we summarize spike and LFP data formats which are supported by the toolboxes. Besides, FieldTrip provides a standard procedure for converting own unsupported data to the FieldTrip structure and Neo-library provides use cases for own data format conversion^11^.

**Table 2:** Spike and LFP data formats which are supported by the open-source spike and LFP data processing and analysis toolboxes.

**Toolbox**	**Blackrock**	**CED**	**Intan**	**MATLAB**	**Plexon**	**Tucker**	**Additionally**
	**(.nev, .nsx)**	**Spike2 (.smr)**	**(.rhd)**	**files (.mat), variables**	**(.pl2, .plx)**	**davis techn**.	
Brainstorm	+	+	+	+	+	+	FieldTrip structures (.mat),
							Neurodata without
							borders (.nwb)
Chronux	−	−	−	+	+	−	−
Elephant and	+	+	+	+	+	+	Neo-supported formats
Spike Viewer							
FieldTrip	+	+	−	+	+	+	MPI (.dap), Windaq (.waq),
							Neuralynx (.ncs, .nse,
							.nts, .nev, .nrd, .dma, .log)
gramm	−	−	−	+	−	−	−
SPIKY	−	−	−	+	−	−	text files (.txt)

*Neo-supported formats include Alpha Omega (.map), Axon (.abf), Igor Pro (.ibw), kwik (.kwik), nest (.dat, .gdf), neuralynx (.nsc, .nse, .nev), Neuroscope, Neuroshare, nix, OpenEphys etc., see the whole list at neo/io/neomatlabio.py*.

### 1.4. Software and Hardware Requirements

All toolboxes from Table 1 are supported by Microsoft Windows, Macintosh and Linux operating system and are easy to install. Considered toolboxes were developed in MATLAB^12^ and Python^13^ languages that are popular in the neuroscientific community. Brainstorm MATLAB version, Chronux, FieldTrip, gramm and SPIKY MATLAB version require MATLAB installation and setting path to the toolbox. Elephant and SPIKY Python version require Python installation and running Python instructions from the command line as specified in documentation^14^^,^^15^. Brainstorm standalone version and Spike Viewer require neither MATLAB nor Python installation, they are installed via GUI-based interface.

As additional software requirements,

- Brainstorm standalone version requires installing freely distributed MATLAB Compiler Runtime^16^. Brainstorm MATLAB version requires MATLAB Signal Processing Toolbox for some functionality;
- Chronux requires MATLAB Signal Processing (for common functionality) and Data Acquisition (for specscope utility) Toolboxes installed. Chronux under Macintosh operating system requires compiling of the locfit (by running locfit/source/compile.m) and spikesort packages;
- Elephant requires installation of Neo and standard numpy, scipy, quantities Python libraries for common functions as well as some libraries for particular packages such as pandas (for using panda_bridge module), scikit-learn (for using ASSET analysis), numpydoc and sphinx (for building documentation) and nose (for running tests);
- FieldTrip requires MATLAB Signal Processing (for filtering and spectral analysis), Statistics and Machine Learning Toolboxes (for spike data analysis and statistical functions);
- gramm requires MATLAB Statistics (for statistical functions) and Curve Fitting (for curve fitting functions) Toolboxes;
- SPIKY MATLAB version requires compiling MEX-files by running SPIKY_compile_MEX.m file. SPIKY does not specify any dependencies on MATLAB toolboxes in the documentation;
- Python Spike Viewer version (compared to standalone version) requires installation of the following Python packages: spykeutils, scipy, guiqwt, matplotlib, tables, spyder, neo (see details at Spike Viewer website).

Hardware dependencies are not specified for the toolboxes besides Brainstorm that requires 64 bit operating system to run efficiently.

### 1.5. Test Dataset

We consider an open-access dataset (Lawlor et al., 2018; Perich et al., 2018) to illustrate each toolboxes' functionality and refer to this dataset as “test dataset.” The dataset contains extracellular recordings from premotor (PMd) and primary motor (M1) cortex from a macaque monkey in a sequential reaching task where monkey controlled a computer cursor using arm movements. A visual cue specified the target location for each reach. The monkey receives a reward after making four correct reaches to the targets within the trial.

In sections 2, 3, we compare toolboxes for the general spike and LFP data analysis, respectively. In section 4, we compare tools for the analysis of synchronization and connectivity in spike and LFP data. Sections 2–4 are each divided into two subsections: first, we compare functionalities common among toolboxes, then we discuss those unique to some toolboxes, i.e., functionality implemented only in one of the toolboxes under comparison. In section 5, we compare toolboxes with specialized tools for dimensionality reduction and generalized linear modeling. Finally, we summarize the comparisons in section 6. In section 7, we list all the considered toolboxes in alphabetical order with links for toolbox downloading and brief descriptions. We do not consider toolboxes specializing on spike sorting and modeling spiking activity in this review. These specializations are referred to in Ince et al. (2010), Mahmud and Vassanelli (2016) and web-reviews^17^^,^^18^^,^^19^ correspondingly.

## 2. Toolboxes for Spike Data Processing and Analysis

In Table 3 we compare major open-source toolboxes for spike data analysis, both for point-process data and for spike waveforms. Functionality related to synchronization and connectivity analysis (e.g., cross-correlation, coherence, joint peri-stimulus time histogram, spike-LFP phase-coupling and dissimilarity measures etc.) will be covered in section 4, and functionality related to dimensionality reduction and generalized linear modeling in section 5.

**Table 3:** Comparing open-source spike data processing and analysis toolboxes.

**Toolbox**	**ISIH**	**PSTH**	**Raster**	**Spike**	**Tuning**	**Statistical**	**Unique features**
			**plots**	**sorting**	**curves**	**tools**	
Brainstorm	−	−	+	+	+	+	−
Chronux	+	+	−	−	−	+	Locfit, MTF
Elephant	+	+	−	−	−	+	CV2, Fano factor, LV
FieldTrip	+	+	+	+	−	+	Waveform statistics
gramm	−	+	+	−	+	+	−
Spike Viewer	+	+	+	−	−	−	−
SPIKY	−	+	+	−	−	−	−

*CV2, measure of inter-spike variability (Holt et al., 1996); ISIH, Inter-Spike Interval Histogram; locfit, local regression and likelihood based analyses (Loader, 2006; Bokil et al., 2010); LV, measure of local variation (Shinomoto et al., 2003); MTF, MultiTaper Fourier transform for point-process data (Jarvis and Mitra, 2001; Bokil et al., 2010); PSTH, Peri-Stimulus Time Histogram*.

In Tables 1, 3 one can see that Brainstorm, Chronux and FieldTrip toolboxes provide more versatile functionality (see also below) than others, are highly cited, well-documented and allow import from many file formats. The Elephant toolbox has versatile functionality (see section 2.2) but it does not have built-in visualization tools (Elephant provides visualization examples in the documentation using matlabplot Python library). Compared to other toolboxes from Table 3,

- Brainstorm and FieldTrip include detailed documentation with tutorials and examples (documentation of other toolboxes from Table 3 has less examples/tutorials for spike data analysis) and have either a forum^20^ or a discussion list^21^ where users can ask questions on data analysis; both toolboxes regularly hold hands-on courses^22^^,^^23^, while other toolboxes from Table 3 provide neither forums nor courses;
- Brainstorm, Elephant and FieldTrip are actively developing by including new functionality;
- FieldTrip provides many descriptive and inferential statistics mostly not requiring MATLAB statistical toolbox (Brainstorm provides statistical tools without examples for spike data analysis^24^. and these statistical functions are not part of spike data analysis functions, different to how it is often done in FieldTrip and Chronux; Spike Viewer and SPIKY do not provide statistical tools for general spike data analysis);
- FieldTrip and gramm allow versatile data plot customization (color maps, line widths, smoothing, errorbars etc.); while gramm provides better and quicker general visualization tools, FieldTrip provides plotting customization specific for spike data analysis (conditions/interval/trials/channels and optimal bin size selection);
- for programming-oriented users, Chronux and FieldTrip provide, to our opinion, most convenient, easy to automatize and well-commented data analysis pipeline with clear uniform data structure (other toolboxes from Table 3 are lacking at least one of three following components: detailed code comments with description of input/output parameters, uniform input/output to the functions throughout the analysis pipeline, modular function design allowing to easily adapt them into analysis workflow). Note that Brainstorm, Spike Viewer, and SPIKY toolboxes assume using a GUI for data analysis. Chronux reference documentation in the function description provides a list of functions which are called from the function and from which the function is called, this is convenient for programming-oriented users.

### 2.1. Comparing Common Tools: Peri-Stimulus Time Histogram, Raster Plot, Inter-Spike Interval Histogram and Spike Sorting

**Figure 1:**
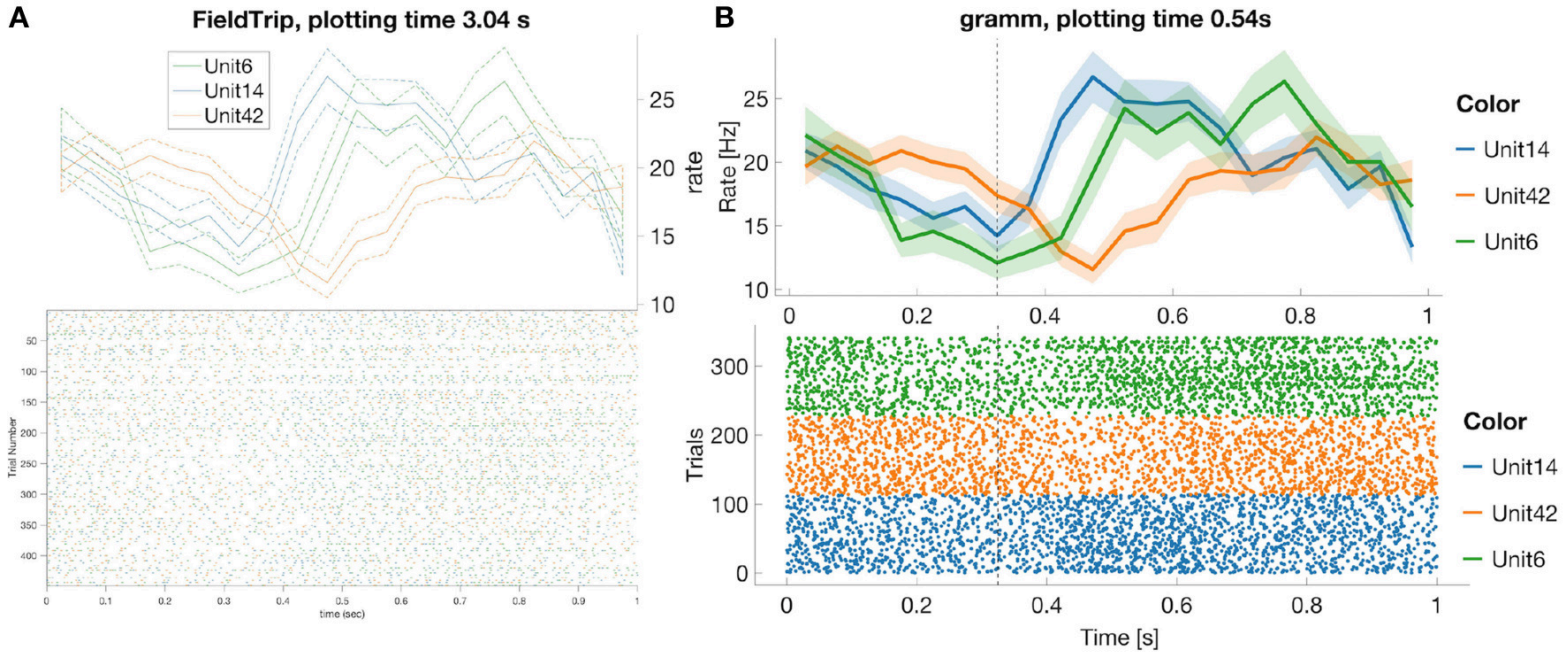
FieldTrip **(A)** and gramm **(B)** provide most adjustable peri-stimulus (PSTH) and raster plots properties (plotting time is averaged over 1,000 runs, MATLAB 2016a, here and later for processor 3.2 GHz Intel Core i5 with 16 GB RAM) among toolboxes from Table 3. We considered 50 ms bin size, M1 units 6, 14, 42, monkey MM for the test dataset. PSTHs are presented with standard error of the mean, neural activity is aligned to trial start for reaches toward the second target in the trial. FieldTrip build-in tools do not allow to adjust font size in a raster plot and line width in a PSTH plot (one has to do it manually with MATLAB tools), and do not allow to plot raster and PSTH in separate figures (though one can plot spike densities in a separate figure). Advantages of gramm toolbox for PSTH and raster plots are quick plotting, raster plots separation for different units, vertical dashed lines for showing event times of the experiment protocol, and smooth adjustment of line width, font size, color maps, errorbar, components positions, etc.

In this subsection we compare most common spike data analysis functions: peri-stimulus time histogram (PSTH), raster plot, inter-spike interval histogram (ISIH) and spike sorting algorithms for toolboxes from Table 3. Regarding visualization, the gramm visualization toolbox stands out with its publication-quality graphics, which helps avoiding major post-processing. This is illustrated in Figure 1, where we compare PSTH and raster plots for test dataset produced in FieldTrip and gramm toolboxes, both of which provide most adjustable plot properties compared to other toolboxes from Table 3 (see below a detailed comparison).

We do not provide raster plots and PSTH plots for other toolboxes from Table 3 with visualization tools since

- Brainstorm does not provide PSTH plots; raster plots are available only for one unit per figure^25^;
- Chronux does not provide raster plots and allows to plot only smoothed PSTH for one unit per figure without built-in tools to adjust line width, font size, colors etc.;
- in SPIKY raster and PSTH plots are available only for one unit per figure (Kreuz et al., 2015, Figure 2) and without confidence intervals for PSTHs;
- in Spike Viewer PSTH plots are available without confidence intervals^26^.

**Figure 2:**
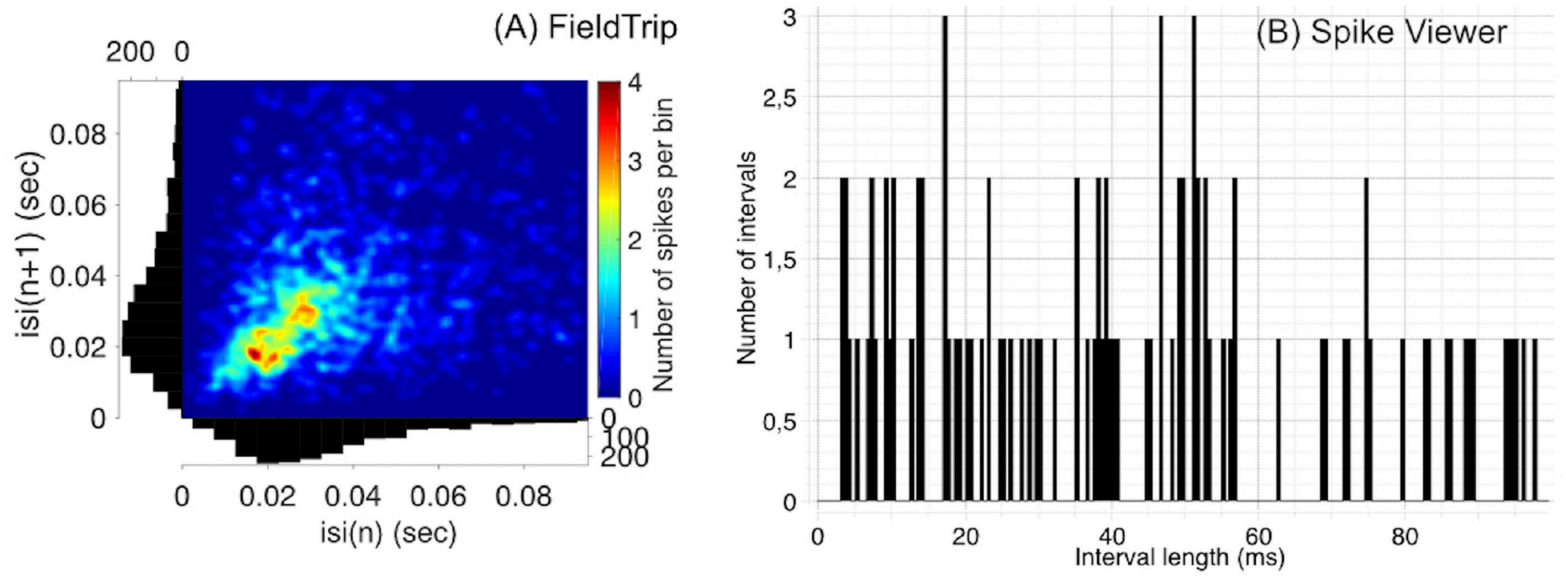
Compared to Spike Viewer **(B)**, FieldTrip **(A)** provides also a second-order statistic on inter-spike interval histogram (ISIH). We considered the test dataset (M1 unit 14 aligned to trial start for reaches toward the first target, monkey MM) for FieldTrip plot and Spike Viewer test dataset for Spike Viewer plot. Font sizes in FieldTrip have been adjusted with MATLAB tools since FieldTrip built-in tools do not provide this option.

Regarding statistical tools when computing PSTHs, Chronux computes PSTH for adaptive or user-defined kernel width with Poisson error or bootstrapped over trials (both with doubled standard deviation error). Elephant computes PSTH for fixed user-defined bin size without additional statistics (note that Elephant provides many kernel functions for convolutions such as rectangular, triangular, Guassian, Laplacian, exponential, alpha function etc.). FieldTrip computes PSTH for optimal (by Scott's formula, Scott, 1979) or user-defined bin width with variance computed across trials. Besides, FieldTrip, different to other toolboxes from Table 3, allows statistical testing on PSTHs for different conditions or subjects^27^ with a parametric statistical or a non-parametric permutation test. Brainstorm provides this functionality by calling FieldTrip functions. gramm allows to compute PSTHs with (bootstrapped) confidence intervals, standard error of the mean, standard deviation etc^28^ only for user-defined bin width. Spike Viewer and SPIKY compute PSTH only for user-defined bin width and do not compute statistics for PSTHs across trials.

In Figure 2 we compare visualization of ISIH provided by FieldTrip and Spike Viewer since other toolboxes from Table 3 do not provide ISIH visualization (Brainstorm, Chronux and Elephant compute ISIH without visualization, see details below).

Regarding statistical tools when computing ISIH, FieldTrip computes ISIH with a coefficient of variation (a ratio of the standard deviation to the mean), Shinomoto's local variation measure (Shinomoto et al., 2005) or a shape scale for a gamma distribution fit. Chronux computes ISIH with two standard deviations away from the mean calculated using jackknife resampling. Elephant computes ISIH with a coefficient of variation. Spike Viewer does not compute statistics on ISIH.

Brainstorm and FieldTrip provide spike sorting algorithms including spike detection and extraction, i.e., using time-continuous broadband data as input. Spike sorting package is no longer provided in Chronux. Brainstorm provides supervised and unsupervised spike sorting according to the methods WaveClus (Quiroga et al., 2004), UltramegaSort2000 (Fee et al., 1996; Hill et al., 2011), KiloSort (Pachitariu et al., 2016), and Klusters (Hazan et al., 2006). FieldTrip implements k-means and Ward (for several Ward distances) sorting methods. While Chronux and FieldTrip do not provide tutorials on spike sorting, Brainstorm has a detailed tutorial^29^.

Brainstorm provides computing and visualization of tuning curves: they are plotted with one figure per unit for selected units, conditions and time interval but without customization of font size, line width and colors, no variance statistic across trials is computed^30^. Gramm toolbox provides visualization of tuning curves including fits from MATLAB curve smoothing toolbox and user-defined functions (also in polar coordinates) with (bootstrapped) confidence intervals, standard error of the mean, standard deviation etc. As the considered gramm version is not focused on spike data analysis, firing rates averaged per condition need to be computed prior to tuning curves visualization (see example in our open MATLAB script).

### 2.2. Description of Unique Tools

In this section we discuss unique tools of toolboxes from Table 3, e.g., fitting tools, and higher order statistics (variability and spectral measures) on spike timing.

Chronux provides two unique tools: local regression package (locfit) and point-process spectrograms. locfit is based on local regression methods (Loader, 2006; Hayden et al., 2009; Parikh, 2009) and provides a set of methods for fitting functions and probability distributions to noisy data. The idea of local regression is that the estimated function is approximated by a low order polynomial in a local neighborhood of any point with polynomial coefficients estimated by the least mean squares method (Bokil et al., 2010). In Bokil et al. (2010) and Loader (2006), local regression methods are motivated by their simplicity, non-parametric approach to kernel smoothing and by reducing the bias at the boundaries which is present in kernel smoothing methods. On the other hand, it was shown that fixed and variable kernel methods (Shimazaki and Shinomoto, 2010, Algorithm 2, Appendix A.2) as well as Abramson's adaptive kernel method (Abramson, 1982) outperform locfit for simulated data examples (Shimazaki and Shinomoto, 2010).

Point-process spectrograms are usually used to illustrate rhythmic properties of otherwise stochastic spiking patterns rather than for statistical inference (Deng et al., 2013). We refer to (Hurtado et al., 2004, 2005) regarding methods to evaluate statistical significance of point-process spectral estimators and to Jarvis and Mitra (2001) and Rivlin-Etzion et al. (2006) for a critical discussion. Chronux provides the only open-source, to our knowledge, implementation of point-process spectral estimates which is implemented according to Jarvis and Mitra (2001) and Rivlin-Etzion et al. (2006, section 4, Formula 11); see an example in our open MATLAB script.

Elephant provides several statistical measures for spike timing variability such as Fano factor, CV2 measure of inter-spike variability (Holt et al., 1996) and a measure of local variation (Shinomoto et al., 2003) which were introduced to overcome the sensitivity of the classical coefficient of variation to firing rate fluctuations (Shinomoto et al., 2005). Elephant also provides a kernel estimation based on Shimazaki and Shinomoto (2010) for calculation of the instantaneous firing rate.

FieldTrip allows to compute mean average spike waveform and its variance across trials, one can optionally align waveforms based on their peaks, rejects outlier waveforms and interpolate the waveforms.

## 3. Toolboxes for LFP Data Analysis

In Table 4 we compare open-source toolboxes for processing and analysis of local field potential (LFP) data. Functionality related to synchronization and connectivity analysis will be discussed in section 4.

**Table 4:** Comparing open-source toolboxes for processing and analysis of LFP data.

**Toolbox**	**Digital**	**De-trending**	**FFT**	**Hilbert**	**Line noise**	**Multitaper**	**Wavelet**	**Statistical**
	**filtering**			**transform**	**removal**	**methods**	**transform**	**tools**
Brainstorm	+	+	+	+	+	+	+	+
Chronux	−	+	+	−	+	+	−	+
Elephant	+	−	+	+	−	−	+	−
FieldTrip	+	+	+	+	+	+	+	+

*FFT, Fast Fourier Transform*.

In Table 4 one can see that Brainstorm and FieldTrip toolboxes provide most versatile functionality for LFP data analysis. Compared to other toolboxes from Table 4,

- FieldTrip provides most flexible and versatile digital filtering (in particular, a fast and accurate line noise removal technique) and spectral analysis tools (see details in section 3.1);
- Brainstorm^31^^,^^32^ and FieldTrip^33^^,^^34^ provide detailed tutorials with guidance on parameter choice and examples for digital filtering and spectral analysis. Chronux provides examples on parameter choice for spectral analysis in manuals^35^ (Pesaran, 2008);
- Brainstorm and Elephant provide fast implementation of Morlet wavelet transform (see details in section 3.1);
- Brainstorm, Chronux and FieldTrip provide statistical tools for computing variance across trials and for comparing between conditions when estimating spectra; Elephant does not compute statistics on the estimated spectra;
- Brainstorm and FieldTrip allow adjustment of plot properties for spectral analysis such as baseline correction, trials and channels selection, colormaps and interactive selection of spectrogram part for further processing. Neither Chronux nor Elephant provide these options. Compared to Brainstorm, FieldTrip also allows to adjust font sizes, titles, plot limits etc.

### 3.1. Comparing Common Tools: Filtering, Detrending, and Spectral Analysis

Digital filtering is implemented in Brainstorm, FieldTrip, and Elephant toolboxes. Compared to toolboxes from Table 4, MATLAB and Python themselves provide more flexible filtering tools. Yet, it is convenient to have filtering within the toolbox pipeline. First, it allows avoiding extra conversion from the toolboxes' format to MATLAB/Python and back. Second, toolboxes allow simplified setting of filter parameters for typical neuroscientific datasets and offer tutorials for their choice for non-experienced users.

Brainstorm, FieldTrip and Elephant toolboxes provide low/high/band-pass and band-stop filters for user-defined frequencies.

- Brainstorm provides Finite Impulse Response (FIR) filters with Kaiser window based on kaiserord functions from MATLAB Signal Processing Toolbox (Octave-based alternatives are used if this toolbox is not available). The user can set 40 or 60 dB stopband attenuation, data are padded with zeros at edges with a half of filter order length (according to the description of the filtering bst_bandpass_hfilter function used by default);
- Elephant provides Infinite Impulse Response (IIR) Butterworth filtering with adjustable order using scipy.signal.filtfilt (with default padding parameters) or scipy.signal.lfilter standard Python functions;
- FieldTrip provides the most flexible filtering tools with user-defined filter type (Butterworth IIR, window sinc FIR filter, FIR filter using either standard MATLAB fir1 or firls function from Signal Processing Toolbox or frequency-domain filter using standard fft and ifft MATLAB functions), padding type and optional parameters such as window type (Hanning, Hamming, Blackman, Kaiser), filter order and direction, transition width, passband deviation, stopband attenuation etc^36^. An automatic tool to deal with filter instabilities (which MATLAB 2016a, to our knowledge, does not provide) is implemented by either recursively reducing filter order or recursively splitting the filter into sequential filters.

**Figure 3:**
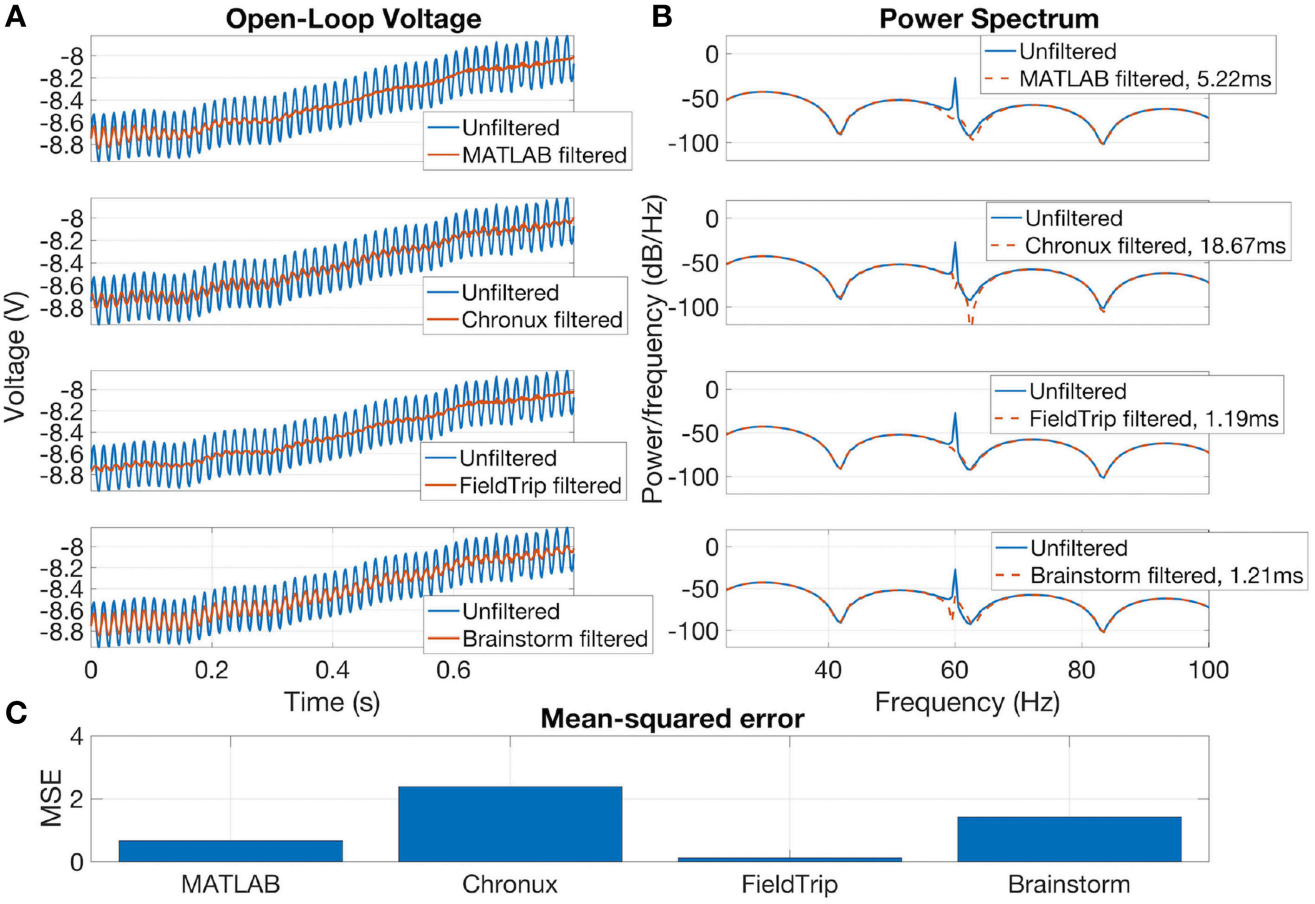
Open-loop voltage **(A)**, Power spectrum **(B)**, and Mean-squared error **(C)** for filtered with open-source toolboxes and unfiltered signal. FieldTrip (discrete Fourier transform filter, default parameters) provides the fastest and the most accurate line noise removal compared to MATLAB solution (Butterworth notch filter with 2 Hz width), Chronux (default 5 tapers, bandwidth 3) and Brainstorm (IIR notch filter with 1 Hz width). Filtering times are averaged over 1,000 runs, MATLAB 2016a.

Brainstorm, Chronux and FieldTrip also provide specific tools for line noise removal. Brainstorm reduces line noise with IIR notch filter (employing either filtfilt function from MATLAB Signal Processing toolbox or MATLAB filter function). Chronux reduces line noise using Thomson's regression method for detecting sinusoids (Thomson, 1982). FieldTrip reduces line noise by two alternative methods: with a discrete Fourier transform (DFT) filter (by fitting a sine and cosine at user-defined line noise frequency and subsequently subtracting estimated components) or by spectrum interpolation (Mewett et al., 2004). In Figure 3 we compare 60 Hz line noise removal by Chronux, FieldTrip and Brainstorm toolboxes on the basis of an example provided by MATLAB^37^ for open-loop voltage across the input of an analog instrument in the presence of 60 Hz power-line noise. One can see that FieldTrip selectively and successfully attenuates 60 Hz while Brainstorm does not fully suppress 60 Hz, Chronux suppresses also frequencies around 62 Hz, the MATLAB solution contains some remaining oscillations in the beginning of the signal, which is also reflected in the periodogram by a slight inaccuracy around 61–62 Hz. In Figure 3 (C) we present mean squared error (MSE) between power spectrum values of the original and estimated signal except the values estimated in 0.2 Hz vicinity of 60 Hz.

Brainstorm, Chronux and FieldTrip provide detrending tools. Brainstorm detrending removes a linear trend from the data, Chronux detrending employs local linear regression^38^, whereas FieldTrip detrending uses a general linear model approach and removes mean and linear trend from the data (by fitting and removing an *N*th order polynomial from the data)^39^. Brainstorm, Chronux and FieldTrip offer similar performance in terms of processing time and trend removal accuracy for a simple MATLAB example^40^ (see our open MATLAB code).

Compared to the classic Fourier transform, multitaper methods provide more convenient control of time and frequency smoothing (Percival and Walden, 1993; Mitra, 2007). Spectral decomposition with Morlet wavelets provides a convenient way of achieving a time-frequency resolution trade-off (van Vugt et al., 2007), since it is inherent to the method that wavelets are scaled in time to vary resolution in time and frequency, see van Vugt et al. (2007) for a comparison of multitaper and wavelet methods and Bruns (2004) for a comparison of wavelet, Hilbert and Fourier transform. Equivalent time-frequency trade-offs can also be implemented with short-time Fourier or Hilbert methods via variable-width tapers (Bruns, 2004).

Chronux and FieldTrip provide multitaper power spectrum estimation using Thomson's method (Thomson, 1982; Percival and Walden, 1993; Mitra and Pesaran, 1999) with Slepian sequences (Slepian and Pollak, 1961). Additionally, FieldTrip allows more conventional tapers (e.g., Hamming, Hanning). In FieldTrip, the user defines frequencies and time interval of interest, width of sliding window and of frequency smoothing. In Chronux, the user defines bandwidth product and number of tapers to be used (see Prieto et al., 2007 for a discussion of multitapers parameter choice).

Brainstorm, Elephant and FieldTrip implement complex-valued Morlet transform. FieldTrip provides time-frequency transformation using Morlet waveforms either with convolution in the time domain or with the multiplication in the frequency domain. Brainstorm and Elephant implement convolution in the time and frequency domain, respectively. FieldTrip implements Morlet wavelet transformation methods based on Tallon-Baudry et al. (1997), the user defines the wavelet width in number of cycles and optionally wavelet length in standard deviations of the implicit Gaussian kernel. In Brainstorm the user sets the central frequency and temporal resolution. Elephant implements Morlet wavelets according to Le Van Quyen et al. (2001) and Farge (1992), where the user sets central Morlet frequencies, size of the mother wavelet and padding type.

Different to other toolboxes in Table 4, FieldTrip also implements Fourier transform on the coefficients of the multivariate autoregressive model estimated with FieldTrip tools (see section 4.1 for more details on MVAR implementation in FieldTrip).

Elephant does not compute statistics on estimated power spectrum whereas Chronux and FieldTrip compute confidence intervals and standard error, correspondingly, in a standard way or with jackknife resampling. To compare spectrum estimates for different conditions or subjects, Chronux provides a two-group test and FieldTrip performs a parametric statistical test, a non-parametric permutation test or a cluster-based permutation test (Brainstorm includes these FieldTrip statistical functions).

MATLAB R2016a, compared to Chronux, FieldTrip and Brainstorm,

- does not provide detailed tutorials for multitaper and wavelet parameters choice;
- does not have built-in tools for computing average spectrogram across trials;
- does not have built-in tools for generating multitaper spectrograms;
- uses exclusively short-time Fourier transform for standard spectrogram plotting.

**Figure 4:**
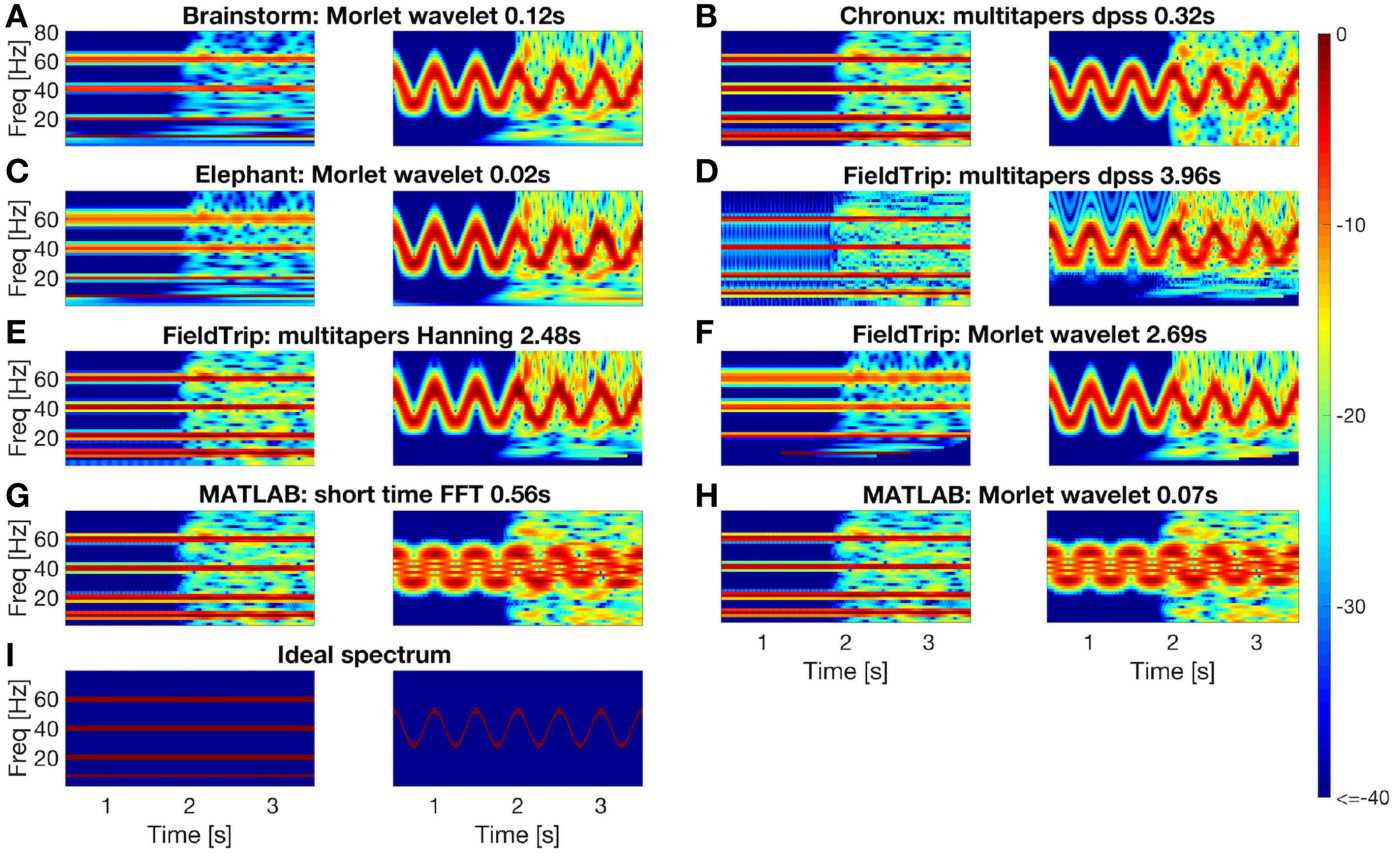
Comparing spectral analysis tools provided by the Brainstorm **(A)**, Chronux **(B)**, Elephant **(C)**, FieldTrip **(D–F)**, MATLAB **(G,H)** compared to ideal spectrum **(I)**. For each toolbox we plot estimated spectrum of signal *x*_1_ (left subpanel) and of signal *x*_2_ (right subpanel). For short-time FFT we used 0.512 s moving window with 0.001s step. For multitaper methods we used in Chronux a single taper with time bandwidth product 2 (left) and 8 (right); in FieldTrip a single taper with 2 Hz (left) and 0.1*F* (right) frequency smoothing for time window 0.512s (left) and 8/*F* (right) at frequency *F*. For wavelet methods we used in MATLAB and Brainstorm central frequency 4 (left) and 1.5 (right) Hz; in Elephant and FieldTrip 20 (left) and 10 (right) cycles wavelets resulting in the spectral bandwidth *F*/10 (left) and *F*/5 (right) Hz at frequency *F*. Spectrum estimating times were averaged over 1,000 runs in MATLAB 2016a.

In Figure 4 we compare spectrum estimation methods implemented in Brainstorm (A), Chronux (B), Elephant (C), FieldTrip (D-F) and MATLAB (G-H) for two simulated signals, *x*_1_(*t*) and *x*_2_(*t*). Note that the results depend on the chosen parameters for each toolbox, see Figure 4 caption for the parameters values.

We generate *x*_1_(*t*) as a sum of sines and *x*_2_(*t*) by sinusoidal frequency modulation, see Equations (1, 2). We add normally distributed pseudo-random values with zero mean to the second half of both signals:

(1) $$\begin{array}{l} x_{1}\left(t\right)=\sin\left(2\pi 8t\right)+\sin\left(2\pi 20t\right)+\sin\left(2\pi 40t\right)+\sin\left(2\pi 60t\right)+\varepsilon \left(t\right) \end{array}$$

(2) $$\begin{array}{l} x_{2}\left(t\right)=\cos2\pi 40t+6\sin\left(2\pi 2t\right)+\varepsilon \left(t\right) \end{array}$$

(3) $$\varepsilon (t)=\{\begin{array}{ll} 0, & \text{for }t=1,2,\ldots ,2000,\\ \sim N(0,1), & \text{for }t=2001,2002,\ldots ,4000, \end{array}$$

where *t* is time in ms.

The instantaneous frequency of the signal *x*_2_(*t*) is defined by the following equation (Granlund, 1949):

(4) $$\begin{array}{l} f\left(t\right)=40+12\cos\left(2\pi 2t\right). \end{array}$$

**Figure 5:**
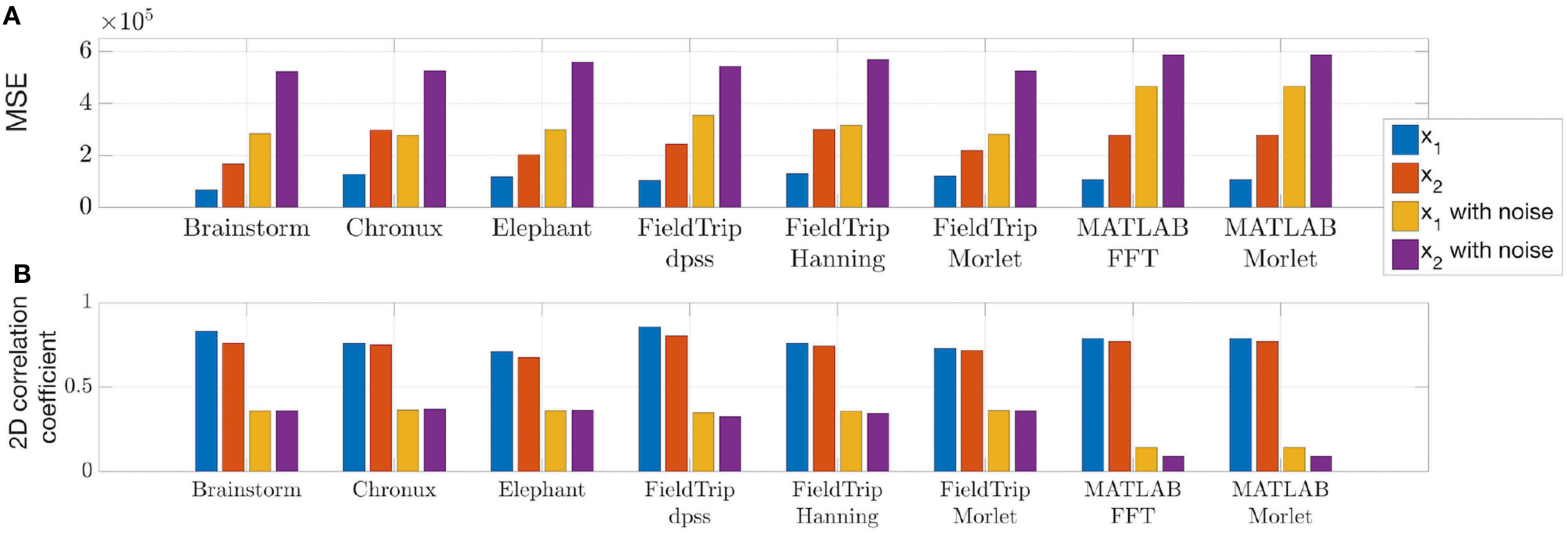
Mean squared error **(A)** and two-dimensional Pearson correlation coefficient **(B)** values between estimated and ideal spectra. These measures were computed for the time span from 1 to 3 s for the signals generated according to Equations (1, 2). The lower MSE and the higher correlation coefficient are, the closer is the estimated spectrum to the ideal spectrum.

To compare quantitatively the spectra estimated by the toolboxes we compute power spectrum values of the ideal signal by setting maximum spectrum values at theoretical frequencies of the signals *x*_1_ (8, 20, 40, and 60 Hz) and *x*_2_ (given by Equation 4) and minimum at all other frequencies. When setting ideal power spectrum values, we allow a bandwidth of 1 Hz, i.e., we set the maximum power spectrum values also at neighboring frequencies. Then in Figure 5 we compare the estimated spectrum values with the ideal spectrum values using mean squared error and two-dimensional Pearson correlation coefficient as suggested in Rankine et al. (2005).

We conclude from Figures 4, 5 that

- MATLAB standard spectrogram tools are less robust with respect to noise than spectrum estimation provided by the toolboxes from Table 4 for the signal *x*_2_ with changing frequencies;
- while Brainstorm, Chronux, Elephant and FieldTrip provide equally good accuracy of spectra estimation, Brainstorm and Elephant provide the fastest computing tools (see spectra computing times in subplot titles of Figure 4).

See in our open MATLAB script an example of spectral analysis with averaging over trials for real-world LFP data (Lowet et al., 2015).

### 3.2. Description of Unique Tools

Compared to other toolboxes from Table 4, Chronux provides several unique features for specialized computations (Bokil et al., 2010) such as space-frequency singular value decomposition (SVD) for univariate and multivariate continuous signals: for theoretical details we refer to Mitra and Pesaran (1999) and for an example of possible application to Makino et al. (2017) and Prechtl et al. (1997). Space-frequency SVD can be applied to the space-time data as, for example, in Prechtl et al. (1997), where space-frequency SVD has been applied for spectral analysis of transmembrane potentials optically recorded in pixels distributed in space. Chronux also provides computation of multitaper spectral derivatives and stationarity statistical test for continuous processes based on quadratic inverse theory.

Elephant provides computing of the current source density from LFP data using electrodes with 2D or 3D geometries (Pettersen et al., 2006; Potworowski et al., 2012).

## 4. Toolboxes With Synchronization and Connectivity Analysis Tools

In Table 5 we compare open-source toolboxes providing tools for spike-spike, field-field (LFP-LFP) or spike-field (spike-LFP) synchronization and connectivity analysis. We refer to Blinowska (2011) and Bastos and Schoffelen (2016) for reviews of functional connectivity analysis methods and their interpretational pitfalls (e.g., common reference, common input, volume conduction or sample size problems). We do not include in Table 5 the connectivity toolboxes ibTB (Magri et al., 2009) and Toolconnect (Pastore et al., 2016), since they are not available under the links provided by the authors (accessed on 27.03.2019). We also do not list in Table 5 the following connectivity analysis toolboxes that are not focused on spike and LFP data analysis: HERMES (Niso et al., 2013), Inform (Moore et al., 2017), JIDT (Lizier, 2014), MVGC (Barnett and Seth, 2014), MuTe (Montalto et al., 2014), PyEntropy (Ince et al., 2009), and TrenTool (Lindner et al., 2011). TrenTool toolbox has a FieldTrip-compatible data structure.

**Table 5:** Comparison of connectivity analysis toolboxes for spike and LFP data.

**Toolbox**	**(Cross)-**	**Coherence**	**Granger**	**Phase-**	**Phase-**	**Spike-**	**Spike-**	**Unique**
	**correlation**		**causality**	**amplitude coupling**	**locking value**	**triggered average**	**field coherence**	**features**
Brainstorm	+	+	+	+	+	+	−	STAT
Chronux	−	+	−	−	−	+	+	STAT
Elephant	+	+	−	−	−	+	+	RSEQ, STD,
								STTC
FieldTrip	+	+	+	+	+	+	+	DTF, JPSTH, MI,
								NC, PDC, PPC,
								PSI, STAT, WPL

*DTF, Directed Transfer Function (Kaminski and Blinowska, 1991); JPSTH, Joint Peri-Stimulus Time Histogram; MI, Mutual Information (Cover and Thomas, 2012); NC, Noise Correlations (Cohen and Kohn, 2011); PDC, Partial Directed Coherence (Baccalá and Sameshima, 2001); PPC, Pairwise Phase Consistency (Vinck et al., 2010); PSI, Phase Sloped Index (Nolte et al., 2004); RSEQ, statistical methods for detected Repeated SEQuences of synchronous spiking (Staude et al., 2010; Torre et al., 2016; Quaglio et al., 2017; Russo and Durstewitz, 2017); STAT, STATistical tools; STD, Spike-Train Dissimilarity measures; STTC, Spike Time Tiling Coefficient (Cutts and Eglen, 2014); WPL, Weighted Phase Lag index (Vinck et al., 2011)*.

Compared to other toolboxes from Table 5,

- Brainstorm, Elephant and FieldTrip provide most versatile set of connectivity measures: while FieldTrip provides many classic and recent pairwise connectivity and synchronization measures, Elephant provides tools for multivariate analysis of high-order correlations in spike trains (see sections 4.1, 4.2);
- Brainstorm tutorials for connectivity measures are actively developing^41^; Chronux has examples for connectivity measures for real-world data in tutorial presentations; FieldTrip provides detailed tutorials on connectivity analysis for simulated and real-world data; Elephant provides examples for connectivity measures with simulated data;
- Chronux and FieldTrip compute confidence intervals for connectivity measures with jackknife resampling or variance estimates across trials, correspondingly (see sections 4.1, 4.2); Brainstorm computes significance values for common connectivity measures, Elephant does not compute statistics on common connectivity measures.

To provide a better feeling of connectivity measures, we classify in Table 6 connectivity and synchronization measures mentioned in Table 5. We indicate for which signals the measure is applicable (Input), whether the measure is directed or not (Directed), is defined in time or frequency domain (Domain) and is bi- or multivariate (Dimension).

**Table 6:** Classification of synchronization and connectivity measures implemented in toolboxes listed in Table 4 regarding whether the measure is directed or not (Directed), is defined in time or frequency domain (Domain) and is bi- or multivariate (Dimension).

**Measure**	**Directed**	**Domain**	**Dimension**
Correlation and cross-correlation (CC)	−	Time	Bivariate
Coherence	−	Frequency	Bivariate
Directed transfer information (DTF)	+	Frequency	Multivariate
Granger causality (GC)	+	Time, frequency	Bivariate
Imaginary part of coherency (iCOH)	−	Frequency	Bivariate
ISI and SPIKE distance, SPIKE synchronization (STD)	−	Time	Bivariate
Joint peri-stimulus time histogram (JPSTH)	−	Time	Bivariate
Mutual information (MI)	−	Time	Bivariate
Noise correlation (NC)	−	Time	Bivariate
Phase amplitude coupling (PAC)	−	Frequency	Bivariate
Partial coherence (pCOH)	+	Frequency	Bivariate
Partial directed coherence (pdCOH)	+	Frequency	Multivariate
Phase-locking value (PLV)	−	Frequency	Bivariate
Pairwise phase consistency (PPC)	+	Frequency	Bivariate
Phase slope index (PSI)	+	Frequency	Bivariate
statistical methods for detecting Repeated SEQuences of	−	Time	Multivariate
synchronous spiking (RSEQ)			
Spike field coherence (SFC)	−	Time	Bivariate
van Rossum and Viktor-Purpura spike train dissimilarity	−	Time	Bivariate
measures (STD)			
Spike time tiling coefficient (STTC)	−	Time	Bivariate
Weighted phase lag index (WPL)	−	Frequency	Bivariate

### 4.1. Comparing Common Tools: Correlation, Cross-Correlation, Coherence, Granger Causality, Phase-Amplitude Coupling, Phase-Locking Value, Spike-Field Coherence and Spike-Triggered Average

In this subsection we compare implementations of common synchronization and connectivity measures for toolboxes from Table 5: correlation, cross-correlation, coherence, Granger causality, phase-amplitude coupling, phase-locking value, spike-field coherence and spike-triggered average.

Brainstorm and Elephant implement correlation, a pairwise non-directional time-domain connectivity measure. Brainstorm computes Pearson correlation coefficient (or optionally covariance) between spike trains and p-value of its significance; correlation is computed equivalently to MATLAB corrcoef function but in a faster vectorized way for *N* > 2 input signals. Elephant computes either Pearson correlation coefficient between binned spike trains (without additional statistics), pairwise covariances between binned spike trains (without additional statistics) or spike time tiling coefficient (STTC) introduced in Cutts and Eglen (2014). STTC, compared to correlation index introduced in Wong et al. (1993), is described as not dependent on signals firing rate, correctly discriminating between lack of correlation and anti-correlation etc. (Cutts and Eglen, 2014). There is also a MATLAB STTC implementation^42^.

Cross-correlation is correlation between two signals computed for different time lags of one signal against the other. Elephant and FieldTrip implement cross-correlation, a pairwise non-directional time-domain connectivity measure. Between two binned spike trains Elephant computes cross-correlation for user-defined window with optional correction of border effect, kernel smoothing (for boxcar, Hamming, Hanning and Bartlett) and normalization. Between two LFP signals Elephant computes the standard unbiased estimator of the cross-correlation function (Stoica and Moses, 2005, Equation 2.2.3) for user-defined time-lags without additional statistics across trials; note that biased estimator of the cross-correlation function is more accurate as discussed in Stoica and Moses (2005). FieldTrip computes cross-correlation between two spike channels for user-defined time lags and bin size (correlogram can optionally be debiased depending on data segment length). FieldTrip computes shuffled and unshuffled correlograms: if two channels are independent, the shuffled cross-correlogram should be the same as unshuffled.

Brainstorm, Chronux, Elephant and FieldTrip implement coherence, a frequency-domain equivalent of cross-correlation (Bastos and Schoffelen, 2016):

- Brainstorm implements coherence according to Carter (1987) computing also *p*-values of parametric significance estimation;
- Chronux computes coherence between two (binned) point-processes or LFP signals using multitaper methos, with confidence intervals or jackknife resampled error bars;
- Elephant computes coherence using Welch's method with phase lags but without additional statistics. Note that for computing coherence across trials in Elephant one has to apply a mean operation on the individual trials coherence values as Elephant does not have a built-in averaging across trials;
- FieldTrip computes coherence according to Rosenberg et al. (1989) with variance estimate across trials. Additionally, FieldTrip provides computing of partial coherence according to Rosenberg et al. (1998), partial directed coherence (Baccalá and Sameshima, 2001) and imaginary part of coherency (Nolte et al., 2004) with variance across trials. Partial directed coherence (PDC) is a directional measure. Compared to coherence, PDC is shown to reflect a frequency-domain representation of the concept of Granger causality (Baccalá and Sameshima, 2001).

Elephant does not provide built-in tools to compare coherence values between two conditions, Chronux provides a two-group test, FieldTrip provides an independent samples Z-statistic via ft_freqstatistics function by the method described in Maris et al. (2007), and Brainstorm is using the FieldTrip ft_freqstatistics function.

Brainstorm and FieldTrip implement Geweke's extension of the original time-domain concept of Granger causality (GC) introduced in Granger (1969) to the frequency domain (Geweke, 1982). GC implemented in Brainstorm and FieldTrip is a frequency-domain pairwise directional measure of connectivity. FieldTrip GC implementation is based on Brovelli et al. (2004). The multivariate autoregressive (MVAR) model in FieldTrip uses biosig or BSMART toolboxes implementation on user choice, which are included in FieldTrip. FieldTrip computes variance of GC values across trials. Neither Brainstorm nor FieldTrip provide built-in tools/prescribed procedure to statistically compare GC values between conditions. Different to FieldTrip, Brainstorm computes as well time-resolved GC between two signals using two Wald statistics according to Geweke (1982) and Hafner and Herwartz (2008). The directed transfer function and partial directed coherence are multivariate extensions of Granger causality (Blinowska, 2011).

**Figure 6:**
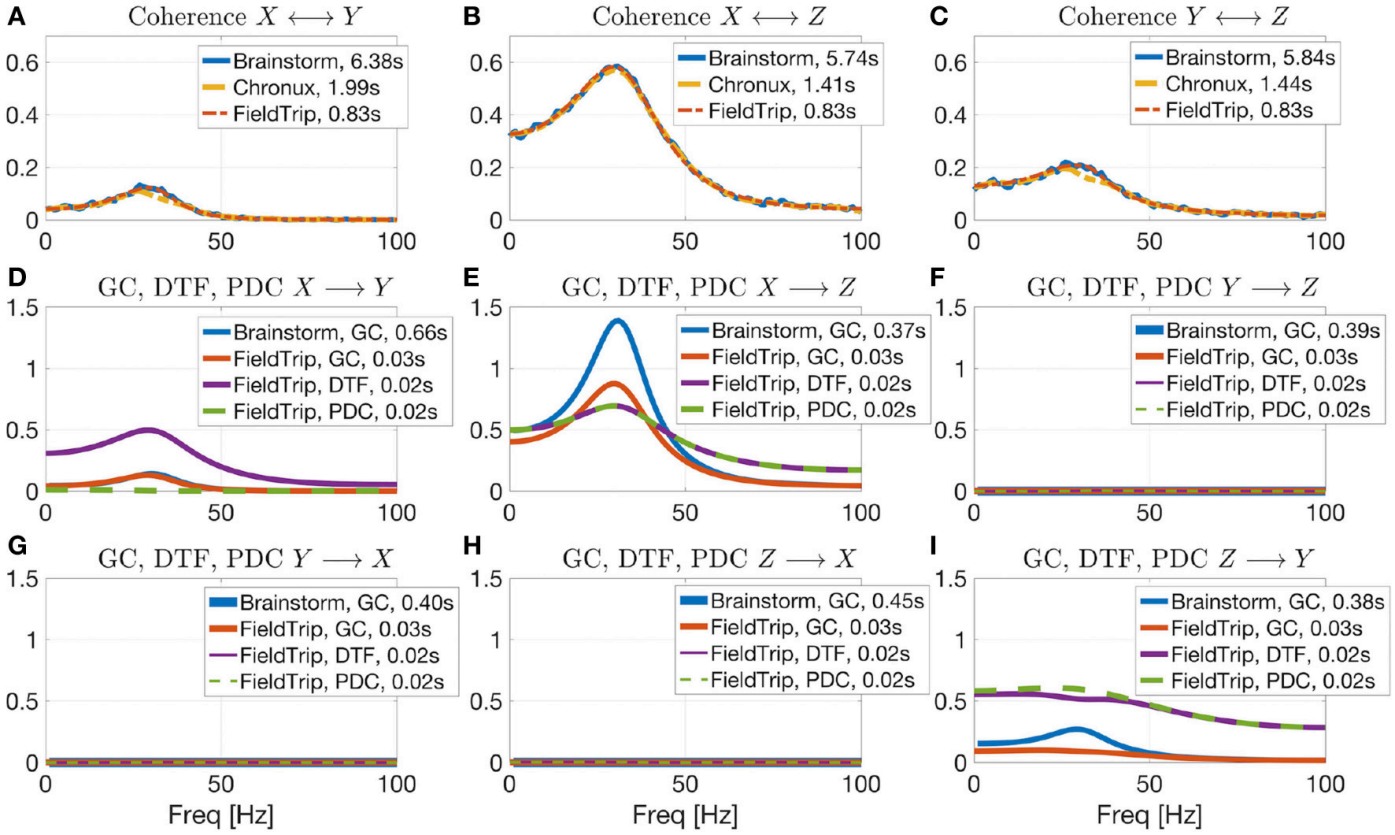
Coherence estimation for *X* ↔ *Y* **(A)**, *X* ↔ *Z* **(B)**, and *Y* ↔ *Z* **(C)** directions and directional measures estimation for *X* → *Y* **(D)**, *X* → *Z* **(E)**, *Y* → *Z* **(F)**, *Y* → *X* **(G)**, *Z* → *X* **(H)**, *Z* → *Y* **(I)** by the open-source connectivity analysis toolboxes. Comparing Brainstorm, Chronux and FieldTrip implementations of connectivity measures for signals simulated by autoregressive models (see Equation 5). While coherence is non-directional, Granger Causality (GC), Directed Transfer Function (DTF, see section 4.2 for more details) and Partial directed Coherence (PDC) are directional measures. PDC allows to correctly detect interaction between signals (note no direct *X*→*Y* interaction). Chronux and FieldTrip provide faster implementations compared to Brainstorm (see somputing times in plots legends) and return variance across trials. Brainstorm coherence values are noisier since there Welch method is used in contrast to multitapers (Chronux) or multivariate autoregressive modeling (FieldTrip).

In Figure 6 we compare values of several connectivity measures computed in Brainstorm, Chronux and FieldTrip for simulated data with autoregressive models^43^ according to Equation (5) (computing coherence across trials is not included in the considered Elephant version). The considered toolboxes show similar pattern of coherence and Granger causality values. Coherence values computed with Brainstorm are noisier than those by Chronux and FieldTrip since Brainstorm employs Welch's method for computing coherence in contrast to multitapers in Chronux and multivariate autoregressive modeling that we used for computing coherence in FieldTrip (using multitapers for spectral decomposition in FieldTrip provides similar results). Note that Brainstorm returns squared coherence values compared to those provided by FieldTrip and Chronux which is why we additionally square them in the code. Note also that we present results for Brainstorm connectivity module for which the tutorial is under development^44^. We also present in Figure 6 the values of Directed Transfer Function (DTF) and Partial Directed Coherence (PDC) to illustrate that PDC correctly detects no interaction between signals in case there is no direct interaction for *X* → *Y* direction (see section 4.2 for more details about DTF and PDC).

(5) $$\begin{array}{l} x(t)=\;0.8x(t-1)-\;0.5x(t-2),\\ y(t)=0.9y(t-1)+\;0.5z(t-1)-0.8y(t-2),\\ z(t)=0.5z(t-1)+\;0.4x(t-1)-0.2z(t-2). \end{array}$$

One of the first steps in the analysis of spike-field coupling is computation of a spike-triggered average (STA) of LFP that is an average LFP voltage within a small window of the time around every spike. While neither Brainstorm nor Elephant compute any additional statistic on STA, Chronux computes STA with an optional kernel smoothing and calculates bootstrapped standard error on computed values and FieldTrip computes mean and variance of STA values.

Brainstorm and FieldTrip implement phase-amplitude coupling (PAC), a frequency-domain pairwise non-directional measure (Canolty et al., 2006; Voytek et al., 2010; Samiee and Baillet, 2017). FieldTrip implements two types of PAC^45^: mean vector length and modulation index according to Tort et al. (2010). Brainstorm implements PAC according to Özkurt and Schnitzler (2011). Both Brainstorm and FieldTrip do not compute additional statistics on PAC.

Brainstorm and FieldTrip implement phase-locking value (PLV), a frequency-domain pairwise non-directional measure (Lachaux et al., 1999). PLV checks how consistent the phase relation between the two signals is across trials. We refer to Vinck et al. (2011) and Bastos and Schoffelen (2016) for a comparison of different phase synchronization metrics and their biases. FieldTrip computes PLV based on (Lachaux et al., 1999) with a variance estimate using jackknife resampling.

The combination of spiking activity and LFP is often used to study rhythmic neuronal synchronization since spike-LFP measures are more sensitive than spike-spike synchronization measures (Vinck et al., 2012; Chakrabarti et al., 2014). To this end, Brainstorm, Chronux, FieldTrip and Elephant implement a spike-field coherence (SFC), a frequency-domain pairwise non-directional measure. Brainstorm implements SFC according to Fries et al. (2001) for user-defined window size around spikes without additional statistics computed. Chronux implements SFC with a multitaper approach for user-defined tapers and frequency band, computing also a confidence level of coherency and jackknife or standard error bars. Elephant implements SFC using standard Python scipy.signal.coherence() function, no additional statistics is computed. FieldTrip computes SFC with variance across trials (see details in the corresponding tutorial^46^).

### 4.2. Description of Unique Tools

In this subsection we describe tools unique to the toolboxes in Table 5. Elephant provides five recent statistical tools to study higher-order correlations and synchronous spiking events in parallel spike trains:

- ASSET (Analysis of Sequences of Synchronous EvenTs) implements the method by Torre et al. (2016) and is an extension of the visualization method by Schrader et al. (2008). ASSET assesses the statistical significance of simultaneous spike events (SSE) and aims to detect such events that cannot be explained on the basis of rate coding mechanisms and arise from spike correlations on shorter time scale;
- CAD (Cell Assembly Detection) implements the method by Russo and Durstewitz (2017) for capturing structures of higher-order correlations in massively parallel spike train recordings with arbitrary time lags and at multiple time-scale; CAD makes statistical parametric testing between each pair of neurons followed by an agglomerative recursive algorithm aiming to detect statistically precise repetitions of spikes in the data;
- CuBIC (Cumulant Based Inference of higher order Correlations) implements a statistical method (Staude et al., 2010) for detecting higher order correlations in parallel spike train recordings;
- SPADE (Spike Pattern Detection and Evaluation) implements the method by Quaglio et al. (2017) for assessing the statistical significance of repeated occurrences of spike sequences (spatio-temporal patterns) based on recent methods in Torre et al. (2013) and Quaglio et al. (2017). SPADE aims to overcome computational and statistical limits in detecting repeated spatio-temporal patterns within massively parallel spike trains (Quaglio et al., 2017); see Quaglio et al. (2018) for a recent review of methods for identification of spike patterns in massively parallel spike trains;
- UE (Unitary Event analysis) implements the statistical method by Grün et al. (1999, 2002) for analyzing excess spike correlations between simultaneously recorded neurons. This method compares the empirical spike coincidences to the expected number on the basis of firing rate of the neurons.

Elephant and SPIKY toolboxes allow to compute measures of spike train dissimilarity (also referred as measures of spike train synchrony). Elephant implements well-known time-scale dependent van Rossum (2001) and Victor and Purpura (1996) dissimilarity distances whereas SPIKY implements three recent parameter-free time-scale independent measures: ISI-distance (Kreuz et al., 2007), SPIKY distance (Kreuz et al., 2012) and SPIKE synchronization (Kreuz et al., 2015). We refer to Chicharro et al. (2011), Kreuz et al. (2012), and Mulansky et al. (2015) for a comparison of dissimilarity measures. Note also MATLAB implementations of dissimilarity measures at J.D. Victor^47^ and T. Kreuz^48^ websites.

FieldTrip, compared to other toolboxes in Table 5, computes and visualizes^49^ the following classic and recent connectivity and synchronization measures:

- Directed Transfer Function (DTF) introduced in Kaminski and Blinowska (1991) is a multivariate frequency-domain directional connectivity measure; FieldTrip computes it according to Kaminski and Blinowska (1991) from cross-spectral density with a variance across trials. DTF, compared to GC, makes a multivariate spectral decomposition which accounts for interaction between all channels (see, e.g., Figure 6 in section 4.1). However, pairwise measures yield more stable results since they involve fitting fewer parameters (Blinowska, 2011; Bastos and Schoffelen, 2016);
- Joint Peri-Stimulus Time Histogram (JPSTH) is a pairwise time-domain non-directional measure between spike trains that allows to gain insight into temporal evolution of spike-spike correlations (Aertsen et al., 1987; Brown et al., 2004). To check whether the resulted JPSTH is caused by task-induced fluctuations of firing rate or by temporal coordination not time-locked to stimulus onset, FieldTrip also computes JPSTH with shuffling subsequent trials;
- Mutual Information (MI) is a pairwise time-domain non-directional connectivity measure. FieldTrip computes MI using implementation from ibtb toolbox (Magri et al., 2009) without additional statistics;
- Noise Correlations (NC) is a non-directional pairwise time-domain measure that can be computed between two spike trains; NC measures whether neurons share trial-by-trial fluctuations in their firing rate; different to so called signal correlations (SC), these fluctuations are measured over repetitions of identical experimental conditions, i.e., are not driven by variable sensory or behavioral conditions;
- phase-coupling pairwise spike-field measures compute the phases of spikes relative to the ongoing LFP with a discrete Fourier transform of an LFP segment around the spike time (Vinck et al., 2012). FieldTrip implements recent methods from Vinck et al. (2012): angular mean of spike phases, Rayleigh p-value and pairwise-phase consistency according to the method in Vinck et al. (2010). We refer to Vinck et al. (2010) and Bastos and Schoffelen (2016) for a discussion and comparison of these measures;
- Phase-Slope Index (PSI) is a directional pairwise frequency-domain measure that can be computed between two signals from their complex-valued coherency. FieldTrip computes PSI according to Nolte et al. (2008) with variance across trials;
- Pairwise Phase Consistency (PPC) is a directional pairwise frequency-domain measure that can be computed from the distribution of pairwise differences of the relative phases. PPC compared to PLV is not biased by sample size (Bastos and Schoffelen, 2016). FieldTrip computes PPC with leave-one-out variance estimate;
- Weighted Phase-Lag index (WPL) introduced in Vinck et al. (2011) is a non-directional pairwise frequency-domain measure computed from cross-spectral density between two signals. WPL was introduced to solve the problem with sensitivity of phase-lag index (Stam et al., 2007) to volume-conduction and noise (Vinck et al., 2011). FieldTrip computes WPL according to Vinck et al. (2011) with variance across trials.

## 5. Specialized Toolboxes for Dimensionality Reduction and Generalized Linear Modeling

In this section we overview specialized toolboxes for dimensionality reduction (section 5.1) and generalized linear modeling (section 5.2). Note that the considered specialized toolboxes do not support importing/exporting from specialized spike data formats and, except DataHigh, they do not have GUI.

### 5.1. Toolboxes for Dimensionality Reduction

Dimensionality reduction of neural data allows to obtain a simplified low-dimensional representation of neural activity. In Table 7 we compare open-source toolboxes for dimensionality reduction of neural data (note also a list of dimensionality reduction software actively updated at B. Yu website^50^). See examples for application of DataHigh, dPCA, and TCA toolboxes in our open MATLAB script.

**Table 7:** Features of open-source dimensionality reduction toolboxes regarding visualization tools, principal and usage programming language, availability of documentation, number of citations, and support by updates at least once per year.

**Toolbox, version**	**Visuali-**	**Language**	**Documen-**	**Cited**	**Support**	**Methods**
	**zation**		**tation**			
DataHigh v.1.2	+	MATLAB	+	<30	In part	FA, GPFA, LDA, PCA
DCA v1.0	−	MATLAB	In part	<30	In part	DCA
		Python				
dPCA v0.1	+	MATLAB	+	<**300**	+	dPCA, PCA
		Python				
GPFA v.2.03	+	MATLAB	In part	>**300**	In part	FA, PCA, pPCA, GPFA
seqNMF	+	MATLAB	+	<30	+	NMF, PCA
tensor-demo	+	MATLAB	+	<30	+	TCA
		Python				
tensortools v0.3.0	+	Python	+	<30	+	ccpTD, nnTCA
TD-GPFA v3.0	+	MATLAB	In part	<30	In part	FA, GPFA, PCA, pPCA

*ccpTD, coupled canonical polyadic Tensor Decomposition; DCA, Distance Covariances Analysis; (GP)FA, (Gaussian Process) Factor Analysis; LDA, Fisher's Linear Discriminant Analysis; NMF, Non-negative Matrix Factorization; (d,p)PCA, (demixed, probabilistic) Principal Component Analysis; (nn)TCA, (non-negative) Tensor Component Analysis. Bold values indicate the number of citations higher than 90*.

We have indicated “In part” in Documentation column for GPFA and TD-GPFA toolboxes since they provide usage examples and readme files with notes on parameters choice but neither detailed manual nor tutorial instead referring to the original publication (Yu et al., 2009) for details. We have indicated “In part” in Documentation column for DCA tool since it also does not provide a manual or tutorial (only example of use in MATLAB script comments). DataHigh and GPFA toolboxes are not uploaded to GitHub or any other public version control system which prevents tracking of version changes and users to submit bug reports. DCA and TD-GPFA toolboxes have not been updated during the last 2 years.

Compared to other toolboxes from Table 7,

- DataHigh provides a user-friendly GUI illustrating algorithm steps such as choice of bin size, smoothing, components number etc.;
- dPCA is applied on trial-averaged spiking activity; dPCA breaks down the neural activity into components each of which relates to time (condition-independent component) or a single experimental condition of the task; the idea is an easier task-relevant interpretation compared to the standard PCA or ICA; the results can be summarized in a single figure (Kobak et al., 2016);
- TD-GPFA allows to extract low-dimensional latent structure from time series in the presence of delays;
- tensor-demo and tensortools allow to reduce dimensionality both across and within trials (Williams et al., 2018).

In Table 8 we outline additional dimensionality reduction tools provided by each toolbox.

**Table 8:** Comparing dimensionality reduction toolboxes: diagnostic and statistical tools.

**Toolbox**	**Cross-validation**	**Tool to select optimal**	**Fitting error**,	**Statistical tests**
		**dimensions number**	**variance explained**	
DataHigh	+	+	+	−
DCA	−	−	−	−
dPCA	+	+	+	+
GPFA	+	+	+	−
seqNMF	+	+	+	+
tensor-demo	−	+	+	−
tensortools	−	+	+	+
TD-GPFA	+	+	+	−

*In Statistical tests column we indicate whether the toolbox provides possibility to measure significance of results and provides permutation or re-shuffling tests on the data*.

It is important to check whether input data fit model assumptions when applying dimensionality reduction methods: whether the data can be non-stationary, contain outliers, observational noise or be correlated, whether recorded activity evolves in a low-dimensional manifold, which sample size is sufficient etc. Discussing model assumptions for each of dimensionality reduction methods is beyond the scope of this paper so we refer to the original papers and to the model assumptions for applying principal component analysis (PCA) formulated in Shlens (2014).

### 5.2. Toolboxes for GLM Analysis

Generalized linear models (GLMs) are often applied for predicting spike counts with the aim to understand which factors influence simultaneous spiking activity: whether it is predicted by the past or concurrent neural activity of the same or remote brain area or by external covariates. In Table 9 we overview major open-source toolboxes for GLM analysis. These toolboxes do not contain any general spike data analysis functions besides GLM analysis since they are either GLM tutorials or codes related to particular analysis made in the paper.

**Table 9:** Features of open-source toolboxes for generalized linear modeling of spike data regarding visualization tools, principal and usage programming language, availability of documentation, number of citations (for the paper with the introduced method), support by updates at least once per year and implemented methods.

**Toolbox, version**	**Methods**	**Visuali-**	**Language**	**Documen-**	**Cited**	**Support**
		**zation**		**tation**		
Case-Studies	pGLM	+	MATLAB	+	<30	+
GLMcode1	pGLM	+	MATLAB	+	<30	−
GLMcode2	pGLM	+	MATLAB	+	<30	−
GLMspiketools v1	cGLM, pGLM, SHF,	+	MATLAB	+	>**900**	+
	STB					
GLMspike-	cGLM, gGLM,	+	MATLAB	+	>**900**	+
traintutorial	pGLM, SHF					
neuroGLM	pGLM, SHF, STB	+	MATLAB	+	>**90**	+
NIMclass v1.0	GLM, GQM, GNM,	−	MATLAB	−	>**90**	+
	NIM					
nStat v2	ppGLM	+	MATLAB	In part	<30	+
spykesML v0.1.dev	pGLM, SHF	−	Python	+	<30	+

*cGLM, GLM with coupling filters; gGLM, linear Gaussian GLM; GNM, Generalized Nonlinear Model (Butts et al., 2011); GQM, Generalized Quadratic Model (Park and Pillow, 2011); pGLM, Poisson GLM (Truccolo et al., 2005); ppGLM, point-process GLM (Paninski et al., 2007); NIM, Nonlinear Input Model (McFarland et al., 2013); SHF, Spikes and covariates History Filters; STB, Smooth Temporal Basis. Bold values indicate the number of citations higher than 90*.

GLMcode1 and GLMcode2 codes are not uploaded to GitHub or any other version control system as they implement methods for particular analysis made in the papers (see below) and are not supposed to be updated.

Note that

- Case-Studies (see folders Chapter 9, 10, 11 on GitHub^51^) implements basic steps of Poisson GLM fitting with history dependence to the data on sample datasets for the corresponding book (Kramer and Eden, 2016);
- GLMcode1, GLMcode2 implement the code for the papers Glaser et al. (2018) and Lawlor et al. (2018);
- examples of use for nStat toolbox are located in helpfiles folder in the corresponding GitHub repository;
- spykesML tool provides comparison of GLM performance with several methods from modern machine learning approaches (including neural networks);
- NIMclass uses MATLAB optimization toolbox and contains many examples for real-world data;
- GLMspiketraintutorial is a tutorial for teaching purposes. It is not memory-efficient implemented, but it makes easy to understand the basic steps of fitting Poisson and Gaussian GLMs, analysis and comparison for spike data^52^. neuroGLM and GLMspiketools are more advanced tools with efficient memory implementation. Additionally to GLMspiketraintutorial, they support some advanced GLM features such as smooth temporal basis functions for spike-history filters, different time-scales for stimulus and spike-history components etc.

## 6. Conclusions

In this review we have compared major open-source toolboxes for spike and local field potentials (LFP) processing and analysis. We have compared toolboxes' functionality, statistical and visualization tools and documentation. Besides summarizing information about toolboxes in comparison tables, we have discussed and illustrated particular toolboxes' functionality and implementations, also in our open MATLAB code. Below we summarize the comparisons that we made for general spike and LFP analysis toolboxes and toolboxes with connectivity tools.

Each considered toolbox has its own advantages:

- Brainstorm: graphical user interface (GUI), versatile and cross-checked functionality (highly-cited), statistical tools, detailed tutorials with recommendations on parameters choice, support of many file formats, active user discussion community and regular hands-on sessions, fast Morlet wavelet transform implementation;
- Chronux: versatile and cross-checked functionality (highly-cited), statistical tools (measures of variance across trials and statistical comparing between different conditions), detailed documentation, convenient data analysis pipeline for programming-oriented users (detailed code comments and modular code design);
- Elephant: support of many file formats, versatile functionality with implementation of classic and recent methods for spike-spike correlation and synchronization analysis, fast Morlet wavelet transform implementation;
- FieldTrip: versatile and cross-checked functionality (highly-cited), statistical tools (measures of variance across trials and statistical comparing between different conditions), detailed tutorials with recommendations on parameters choice, support of many file formats, active user discussion community and regular hands-on sessions, flexible visualization tools, convenient data analysis pipeline for programming-oriented users (detailed code comments and modular code design), versatile filtering, connectivity and synchronization analysis tools, fast and accurate line noise removal;
- gramm: quick publication-quality PSTH, raster plots and tuning curves with many easily adjustable plot properties;
- Spike Viewer: GUI, support of many file formats;
- SPIKY: GUI, implementation of recent spike train dissimilarity measures.

## 7. List of Toolboxes and Tools in Alphabetical Order With Links

Below all the considered toolboxes are provided with a brief description, reference to the paper where the toolbox was introduced and a link for downloading.

- Brainstorm^53^^,^^54^ (Tadel et al., 2011) – a MATLAB toolbox for the analysis of brain recordings: MEG, EEG, fNIRS, ECoG, depth electrodes and animal invasive neurophysiology;
- BSMART^55^ (Brain-System for Multivariate AutoRegressive Time series) (Cui et al., 2008) – a MATLAB/C toolbox for spectral analysis of continuous neural data recorded from several sensors;
- Case-Studies^56^ – a MATLAB set of examples on sample datasets accompanying the corresponding book (Kramer and Eden, 2016);
- Chronux^57^ (Bokil et al., 2010) – a MATLAB package for the analysis of neural data;
- cSPIKE^58^ (Kreuz et al., 2017) – a MATLAB toolbox for computing ISI-distance, SPIKE-distance, SPIKE synchronization and their adaptive variants as well as basic plot functions for plotting spike trains and profiles;
- DataHigh^59^ (Cowley et al., 2013) – a MATLAB-based graphical user interface to visualize and interact with high-dimensional neural population activity;
- DATA-MEAns^60^ (Bonomini et al., 2005) – a Delphi7 tool for the classification and management of neural ensemble recordings;
- DCA^61^ (Cowley et al., 2017) (distance covariance analysis) – an implementation (MATLAB and Python) of the linear dimensionality reduction method that can identify linear and nonlinear relationships between multiple datasets;
- dPCA^62^ (demixed Principal Component Analysis) (Kobak et al., 2016) – a MATLAB implementation of the linear dimensionality reduction technique that automatically discovers and highlights the essential features of complex population activities;
- Elephant^63^^,^^64^ (Yegenoglu et al., 2017) – an Electrophysiology Analysis Toolkit in Python. Elephant toolbox includes functionality from earlier developed toolboxes CSDPlotter^65^ (Pettersen et al., 2006) and iCSD 2D^66^, it is a direct successor of NeuroTools;
- FieldTrip^67^^,^^68^ (Oostenveld et al., 2011) – a MATLAB toolbox for advanced analysis of MEG, EEG, and invasive electrophysiological (spike and LFP) data;
- FIND^69^ (Meier et al., 2008) – a MATLAB toolbox for the analysis of neuronal activity;
- GLMcode1 – a MATLAB code implementing data analysis for particular publication (Glaser et al., 2018) with GLM fitting to analyze factors contributing to neural activity (this code is available from the authors upon request);
- GLMcode2^70^ (Perich et al., 2018) – a MATLAB code implementing data analysis for particular publication (Lawlor et al., 2018) with GLM fitting to estimate preferred direction for each neuron;
- GLMspikestools^71^ (Pillow et al., 2008) – a Generalized Linear Modeling tool for single and multi-neuron spike trains;
- GLMspiketraintutorial^72^ (Pillow et al., 2008) – a simple tutorial on Gaussian and Poisson GLMs for single and multi-neuron spike train data;
- GPFA^73^ (Gaussian-Process Factor Analysis) (Yu et al., 2009) – a MATLAB implementation of the method extracting low-dimensional latent trajectories from noisy, high-dimensional time series data. It combines linear dimensionality reduction (factor analysis) with Gaussian-process temporal smoothing in a unified probabilistic framework;
- gramm^74^^,^^75^ (Morel, 2018) – a plotting MATLAB toolbox for quick creation of complex publication-quality figures;
- HERMES^76^ (Niso et al., 2013) – a MATLAB toolbox for assessing connectivity and synchronization between time series;
- ibTB^77^ (Information Breakdown Toolbox) (Magri et al., 2009) – a C/MATLAB toolbox for fast information analysis of multiple-site LFP, EEG and spike train recordings;
- Inform^78^ (Moore et al., 2017) – a cross-platform C library for information analysis of dynamical systems;
- infoToolbox^79^ (Magri et al., 2009) – a toolbox for the fast analysis of multiple-site LFP, EEG and spike train recordings;
- JIDT^80^ (Lizier, 2014) – an information-theoretic Java toolbox for studying dynamics of complex systems;
- MEAbench^81^ (Wagenaar et al., 2005) – a C++ toolbox for multi-electrode data acquisition and online analysis;
- MEA-tools^82^ (Egert et al., 2002) – a collection of MATLAB-based tools to analyze spike and LFP data from extracellular recordings with multi-electrode arrays;
- MuTe^83^ (Montalto et al., 2014) – a MATLAB toolbox to compare established and novel estimators of the multivariate transfer entropy;
- MVGC^84^ (Multivariate Granger Causality MATLAB Toolbox) (Barnett and Seth, 2014) – a MATLAB toolbox facilitating Granger-causal analysis with multivariate multi-trial time series data;
- neuroGLM^85^ (Park et al., 2014) – an MATLAB tool, an extension of GLMspiketraintutorial allowing more advanced features of GLM modeling such as smooth basis functions for spike-history filters, memory-efficient temporal convolutions, different timescales for stimulus and spike-history components, low-rank parametrization of spatio-temporal filters, flexible handling of trial-based data;
- NIMclass^86^^,^^87^ (McFarland et al., 2013) – a MATLAB implementation of the nonlinear input model. In this model, the predicted firing rate is given as a sum over nonlinear inputs followed by a “spiking nonlinearity” function;
- nStat^88^ (neural Spike Train Analysis Toolbox) (Cajigas et al., 2012) – an object-oriented MATLAB toolbox that implements several models and algorithms for neural spike train analysis;
- OpenElectrophy^89^^,^^90^ (Garcia and Fourcaud-Trocmé, 2009) – a Python framework for analysis of intro- and extra-cellular recordings;
- PyEntropy^91^ (Ince et al., 2009) – a Python module for estimating entropy and information theoretic quantities using a range of bias correction methods;
- PySpike^92^ (Kreuz et al., 2015; Mulansky et al., 2015) – a Python library computing the ISI-distance, SPIKE-distance as well as SPIKE-Synchronization;
- seqNMF^93^ (Mackevicius et al., 2019) – a MATLAB toolbox for unsupervised discovery of temporal sequences in high-dimensional datasets with applications to neuroscience;
- SigMate^94^ (Mahmud et al., 2012) – a MATLAB toolbox for extracellular neuronal signal analysis;
- sigTOOL^95^ (Lidierth, 2009) – a MATLAB toolbox for spike data analysis;
- Spike Viewer^96^^,^^97^ (Pröpper and Obermayer, 2013) – a multi-platform GUI application for navigating, analyzing and visualizing electrophisiological datasets;
-SPIKY^98^ (Kreuz et al., 2015; Mulansky et al., 2015) – a MATLAB graphical user interface that facilitates application of time-resolved measures of spike-train synchrony to both simulated and real data;
- SPKTool^99^ (Liu et al., 2011) – a MATLAB toolbox for spikes detection, sorting and analysis;
- spykesML^100^ (Benjamin et al., 2018) – a Python toolbox with a tutorial for comparing performance of GLM with modern machine-learning methods (neural networks, random forest etc.);
- STAR^101^ (Spike Train Analysis with R) (Pouzat and Chaffiol, 2009) – an R package to analyze spike trains;
- STAToolkit^102^ (Spike Train Analysis Toolkit) (Goldberg et al., 2009) – a MATLAB package for the information theoretic analysis of spike train data;
- tensor-demo^103^ – a MATLAB and Python package (available for both languages) for fitting and visualizing canonical polyadic tensor decompositions of higher-order data arrays;
- tensortools^104^ – a Python package for fitting and visualizing canonical polyadic tensor decompositions of higher-order data arrays;
- TD-GPFA^105^ (time-delayed Gaussian-Process Factor Analysis) (Lakshmanan et al., 2015) – a MATLAB implementation of GPFA method extension that allows for a time delay between each latent variable and each neuron;
- ToolConnect^106^ (Pastore et al., 2016) – a functional connectivity C# toolbox with GUI for *in vitro* networks;
- Trentool^107^ (Lindner et al., 2011) – a MATLAB toolbox for the analysis of information transfer in time series data. Trentool provides user friendly routines for the estimation and statistical testing of transfer entropy in time series data.

## Author Contributions

VU performed the reported study and wrote the paper. AG edited the paper. VU and AG have seen and approved the final manuscript.

### Conflict of Interest Statement

The authors declare that the research was conducted in the absence of any commercial or financial relationships that could be construed as a potential conflict of interest.

## Footnotes

^1^https://github.com/ValentinaUn/Testing-open-source-toolboxes

^2^according to Google Scholar in March 2019 (https://scholar.google.com)

^3^when the code is available under a license which allows free redistribution and the creation of derived works

^4^https://github.com/OpenElectrophy/OpenElectrophy

^5^https://github.com/piermorel/gramm/blob/master/gramm%20cheat%20sheet.pdf

^6^https://elephant.readthedocs.io/en/latest/tutorial.html

^7^https://github.com

^8^https://github.com/NeuralEnsemble/python-neo

^9^http://neuralensemble.org/neo/

^10^see https://neuroimage.usc.edu/brainstorm/Introduction, Chronux folder dataio and http://www.fieldtriptoolbox.org/dataformat for details, correspondingly

^11^https://neo.readthedocs.io/en/0.7.1/usecases.html

^12^https://www.mathworks.com

^13^https://www.python.org

^14^https://elephant.readthedocs.io/en/latest/install.html

^15^https://github.com/mariomulansky/PySpike

^16^https://neuroimage.usc.edu/brainstorm/Installation

^17^https://simonster.github.io/SpikeSortingSoftware/

^18^https://www.cnsorg.org/software

^19^https://grey.colorado.edu/emergent/index.php/Comparison_of_Neural_Network_Simulators

^20^https://neuroimage.usc.edu/forums/

^21^http://www.fieldtriptoolbox.org/discussion_list/

^22^http://www.fieldtriptoolbox.org/workshop/

^23^https://neuroimage.usc.edu/brainstorm/Training

^24^https://neuroimage.usc.edu/brainstorm/Tutorials/Statistics

^25^https://neuroimage.usc.edu/brainstorm/e-phys/functions

^26^https://spyke-viewer.readthedocs.io/en/latest/

^27^http://www.fieldtriptoolbox.org/reference/ft_timelockstatistics/

^28^https://github.com/piermorel/gramm/blob/master/gramm

^29^https://neuroimage.usc.edu/brainstorm/e-phys/SpikeSorting?highlight=%28sorting%29

^30^https://neuroimage.usc.edu/brainstorm/e-phys/functions

^31^https://neuroimage.usc.edu/brainstorm/Tutorials/ArtifactsFilter

^32^https://neuroimage.usc.edu/brainstorm/Tutorials/TimeFrequency

^33^http://www.fieldtriptoolbox.org/example/determine_the_filter_characteristics/

^34^http://www.fieldtriptoolbox.org/tutorial/timefrequencyanalysis/

^35^http://chronux.org

^36^http://www.fieldtriptoolbox.org/reference/ft_preprocessing

^37^https://www.mathworks.com/help/signal/ug/remove-the-60-hz-hum-from-a-signal.html

^38^http://chronux.org/chronuxFiles//Documentation/chronux/spectral_analysis/continuous/locdetrend.html

^39^http://www.fieldtriptoolbox.org/reference/ft_preproc_detrend/

^40^https://de.mathworks.com/help/matlab/data_analysis/detrending-data.html

^41^https://neuroimage.usc.edu/brainstorm/Tutorials/Connectivity

^42^https://github.com/Leo-GG/NeuroFun/blob/master/%2Bcorrel/calcSTTC.m

^43^http://www.fieldtriptoolbox.org/tutorial/connectivity/

^44^https://neuroimage.usc.edu/brainstorm/Tutorials/Connectivity

^45^http://www.fieldtriptoolbox.org/reference/ft_crossfrequencyanalysis/

^46^http://www.fieldtriptoolbox.org/tutorial/spikefield/

^47^http://www-users.med.cornell.edu/~jdvicto/pubalgor.html

^48^http://wwwold.fi.isc.cnr.it/users/thomas.kreuz/Source-Code/VanRossum.html

^49^http://www.fieldtriptoolbox.org/reference/ft_connectivityplot/

^50^http://users.ece.cmu.edu/~byronyu/software.shtml

^51^https://github.com/Mark-Kramer/Case-Studies-Kramer-Eden

^52^https://github.com/pillowlab/GLMspiketraintutorial

^53^https://neuroimage.usc.edu/brainstorm/Introduction

^54^https://github.com/brainstorm-tools/brainstorm3

^55^http://www.brain-smart.org

^56^https://github.com/Mark-Kramer/Case-Studies-Kramer-Eden

^57^http://chronux.org

^58^http://wwwold.fi.isc.cnr.it/users/thomas.kreuz/Source-Code/cSPIKE.html

^59^http://users.ece.cmu.edu/~byronyu/software/DataHigh/datahigh.html

^60^http://cortivis.umh.es

^61^https://github.com/BenjoCowley/dca

^62^https://github.com/machenslab/dPCA

^63^http://python-elephant.org

^64^https://github.com/NeuralEnsemble/elephant/commits/master

^65^https://github.com/espenhgn/CSDplotter

^66^http://www.neuroinf.pl/Members/szleski/csd2d/toolbox

^67^http://www.fieldtriptoolbox.org

^68^https://github.com/fieldtrip/fieldtrip

^69^http://find.bccn.uni-freiburg.de

^70^https://crcns.org/data-sets/motor-cortex/pmd-1/about-pmd-1

^71^http://pillowlab.princeton.edu/code_GLM.html

^72^https://github.com/pillowlab/GLMspiketraintutorial

^73^http://users.ece.cmu.edu/~byronyu/software.shtml

^74^https://de.mathworks.com/matlabcentral/fileexchange/54465-gramm-complete-data-visualization-toolbox-ggplot2-r-like

^75^https://github.com/piermorel/gramm

^76^http://hermes.ctb.upm.es

^77^http://www.ibtb.org

^78^https://github.com/ELIFE-ASU/Inform

^79^http://www.infotoolbox.org

^80^https://github.com/jlizier/jidt

^81^http://www.danielwagenaar.net/meabench.html

^82^http://material.brainworks.uni-freiburg.de/research/meatools/

^83^https://figshare.com/articles/MuTE_toolbox_to_evaluate_Multivariate_Transfer_Entropy/1005245Entropy/1005245

^84^http://www.sussex.ac.uk/sackler/mvgc/

^85^https://github.com/pillowlab/neuroGLM

^86^http://neurotheory.umd.edu/nimcode

^87^https://github.com/dbutts/NIMclass

^88^https://github.com/iahncajigas/nSTAT

^89^http://neuralensemble.org/OpenElectrophy/

^90^https://github.com/OpenElectrophy/OpenElectrophy

^91^https://github.com/robince/pyentropy

^92^https://github.com/mariomulansky/PySpike

^93^https://github.com/FeeLab/seqNMF

^94^https://sites.google.com/site/muftimahmud/codes

^95^http://sigtool.sourceforge.net/

^96^https://github.com/rproepp/spykeviewer

^97^https://spyke-viewer.readthedocs.io/en/latest/

^98^http://wwwold.fi.isc.cnr.it/users/thomas.kreuz/Source-Code/SPIKY.html

^99^https://sourceforge.net/projects/spktool/files/latest/download

^100^https://github.com/KordingLab/spykesML

^101^https://sites.google.com/site/spiketrainanalysiswithr/

^102^https://omictools.com/statoolkit-tool

^103^https://github.com/ahwillia/tensor-demo

^104^https://github.com/ahwillia/tensortools

^105^https://github.com/karts25/NeuralTraj

^106^http://software.incf.org/software/toolconnect

^107^https://trentool.github.io/TRENTOOL3/
